# Population dynamics and genetic changes of *Picea abies *in the South Carpathians revealed by pollen and ancient DNA analyses

**DOI:** 10.1186/1471-2148-11-66

**Published:** 2011-03-10

**Authors:** Enikő K Magyari, Ágnes Major, Miklós Bálint, Judit Nédli, Mihály Braun, István Rácz, Laura Parducci

**Affiliations:** 1 MTA-MTM Research Group for Paleontology, 1476 Budapest, P. O. Box 222, Hungary; 2 Hungarian Natural History Museum, 1431 Budapest, P. O. Box 137, Hungary; 3 Molecular Biology Center, Babeş -Bolyai University, Str. Treboniu Laurian 42, 400271 Cluj, Romania; 4 Balaton Limnological Research Institute, 8237 Tihany, P.O. Box 35, Hungary; 5 University of Debrecen, Department of Inorganic and Analytical Chemistry, 4010 Debrecen, P.O. Box 21, Hungary; 6 Department of Ecology and Genetics, Evolutionary Biology Centre, Uppsala University, Norbyvägen 18D, 75236, Uppsala, Sweden; 7 Biodiversität und Klima Forschungszentrum (BiK-F), Senckenberganlage 25, D-60325 Frankfurt am Main, Germany

## Abstract

**Background:**

Studies on allele length polymorphism designate several glacial refugia for Norway spruce (*Picea abies*) in the South Carpathian Mountains, but infer only limited expansion from these refugia after the last glaciation. To better understand the genetic dynamics of a South Carpathian spruce lineage, we compared ancient DNA from 10,700 and 11,000-year-old spruce pollen and macrofossils retrieved from Holocene lake sediment in the Retezat Mountains with DNA extracted from extant material from the same site. We used eight primer pairs that amplified short and variable regions of the spruce cpDNA. In addition, from the same lake sediment we obtained a 15,000-years-long pollen accumulation rate (PAR) record for spruce that helped us to infer changes in population size at this site.

**Results:**

We obtained successful amplifications for Norway spruce from 17 out of 462 pollen grains tested, while the macrofossil material provided 22 DNA sequences. Two fossil sequences were found to be unique to the ancient material. Population genetic statistics showed higher genetic diversity in the ancient individuals compared to the extant ones. Similarly, statistically significant Ks and Kst values showed a considerable level of differentiation between extant and ancient populations at the same loci.

Lateglacial and Holocene PAR values suggested that population size of the ancient population was small, in the range of 1/10 or 1/5 of the extant population. PAR analysis also detected two periods of rapid population growths (from *ca*. 11,100 and 3900 calibrated years before present (cal yr BP)) and three bottlenecks (around 9180, 7200 and 2200 cal yr BP), likely triggered by climatic change and human impact.

**Conclusion:**

Our results suggest that the paternal lineages observed today in the Retezat Mountains persisted at this site at least since the early Holocene. Combination of the results from the genetic and the PAR analyses furthermore suggests that the higher level of genetic variation found in the ancient populations and the loss of ancient allele types detected in the extant individuals were likely due to the repeated bottlenecks during the Holocene; however our limited sample size did not allow us to exclude sampling effect.

This study demonstrates how past population size changes inferred from PAR records can be efficiently used in combination with ancient DNA studies. The joint application of palaeoecological and population genetics analyses proved to be a powerful tool to understand the influence of past population demographic changes on the haplotype diversity and genetic composition of forest tree species.

## Background

In the last two decades ancient DNA (aDNA) has been successfully extracted from fossil plant material, and the results from the specimens of late Quaternary remains (up to 100 ka yrs) have provided insights into many evolutionary processes [1-9]. These studies represented the first attempts in linking extant and fossil plant populations and provided important information on genetic changes through time. Parducci *et al. *[7] did a promising attempt to analyze short chloroplast DNA (cpDNA) regions in fossil pollen extracted from a Holocene lake sediment in Sweden. Fossil pollen grains are abundant in lake sediments. Under ideal preservation conditions (neutral pH, low temperature, no oxygen) the study of pollen DNA allows studying past demographic events by analyzing neutrally evolving regions of the organelle genome, such as microsatellites and introns [10,11]. During the Quaternary (last 2.6 million years) climatic oscillations have dramatically influenced the distribution of plant species [12]. Repeated range expansions and contractions in response to warming and cooling resulted in large-scale demographic changes with consequent impact on their genetic composition [11].

Progress in aDNA-based phylogenetic and population genetic studies have been very rapid in animal species and lately several specific primers have been designed to study population-level changes (reviewed in Leonard [13] and Hofreiter [14]). Rohland *et al. *[15] have for example detected divergence events between mammoth and the two extant elephant species. Barnes *et al. *[16] and Valdiosera *et al. *[17] successfully reconstructed population movements during the last glaciation in brown bears and detected frequent events of extinctions and recolonizations. Unfortunately, similar studies on woody plants are still in their infancy with few attempts done on fossil pollen and wood [7,18,19]. This is despite the many questions that plant aDNA analyses are amenable to answer [20].

In this study we focus on *Picea abies *(Norway spruce), one of the ecologically and economically most important forest tree species in Europe. Pollen and macrofossil evidence combined with genetic surveys of the current European populations of this species showed that its wide-ranging distribution in central and south-eastern Europe originated from several glacial refugia [21-26]. One such refugium was identified in the South Carpathians, where several unique cpDNA and mitochondrial DNA (mtDNA) haplotypes suggested long-lasting isolation [22,25]. Populations from these regions showed lower level of genetic variation compared to other refugial populations in central Europe, and north- and westward postglacial expansion from this area showed to be limited, as these haplotypes do not appear in the central and northwest European populations today. In addition, the genetic diversity increased north of the Carpathians, probably as a result of admixture of expanding populations from two separate refugia. Tollefsrud *et al. *[25] suggested that the dry plains of central and south-eastern Europe, including the Hungarian Plain, formed an ecological barrier to any expansion, whereas humid mountain ranges elsewhere in central Europe facilitated the northward spread of other spruce lineages. The lateglacial and early Holocene warming also brought major altitudinal displacements of the species range in the South Carpathians [27,28]. In addition, fossil pollen records indicate that a large proportion of glacial lowland coniferous woodlands went destroyed by fire in the early Holocene as summer temperatures rose [29]. An ongoing pollen and plant macrofossil study of a sediment sequence retrieved from a lake located in the Retezat Mountains (Tăul dintre Brazi) in the South Carpathian Range demonstrates that at the onset of the early Holocene warming (around 11,000 cal yr BP) a rapid population expansion of Norway spruce took place around this lake [30].

Based on all this knowledge, we hypothesized two possible population demographic scenarios during the Holocene in the South Carpathian refugium. (1) The spruce populations of the Retezat Mountains remained constrained to this region with a relatively constant population size. In this case we expect to find the same haplotypes and similar levels of diversity in extant and ancient samples. (2) The population of the Retezat Mts underwent significant demographic changes, most probably due to climatic shifts and anthropogenic influences. In this case we expect altered haplotype frequencies in the extant population relative to the ancient population, and different levels of genetic diversity.

**Figure 1:**
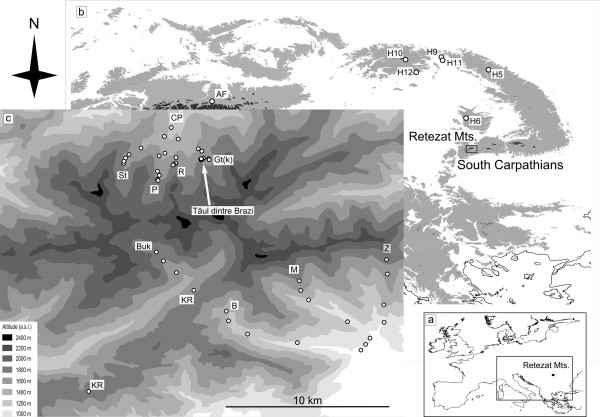
**Sampling sites in the Retezat Mountains**. (a) Map showing the location of the Retezat Mountains in Europe and (b) in the Carpathians; H: herbarium sample; AF: Austrian Alps. (c) Relief map of the Retezat Mts showing the position of Tăul dintre Brazi lake with location of the extant samples analysed in this study. The location of herbarium specimens is shown in map (b). On map (c) labels mark valleys and tracks in the Retezat Mountains; CP: Carnic-Pietrele; St: Valea Stanisoarei; P: Valea Pietrele; R: Vale Rea; Gt(k): Valea Gales; Buk: Valea Bucura; KR: Rtezatul Mic; B: Buta; M. Valea Marii; Z: Vale Zanoaga.

**Figure 2:**
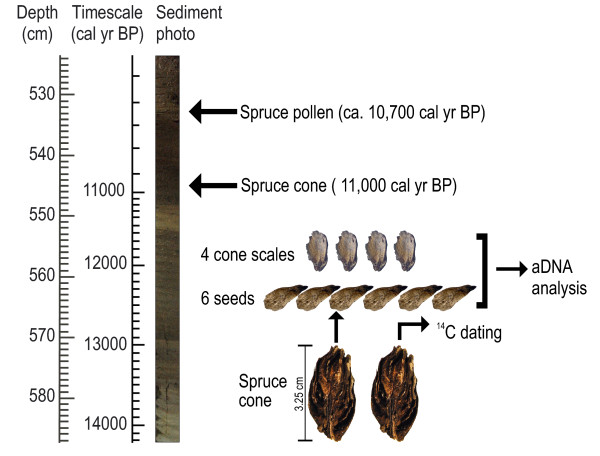
**Sample location and schematics of the core TDB-1 (Tăul dintre Brazi) with a digital photo of the sediment where the ancient spruce samples were collected**. On the left, depth and calibrated BP timescales are shown.

To asses the consistency of the two scenarios, we analyzed fossil spruce pollen and other macrofossils (seeds and cone scales) from the early Holocene section of a sediment core extracted at lake Tăul dintre Brazi (TDB) in the Retezat Mountains (Figure 1). We analyzed cpDNA from material retrieved from two radiocarbon dated sediment layers (11,000 and 10,700 cal yr BP levels) (Figure 2) and compared results with genetic data obtained from the extant spruce population of the Retezat Mountains. Population genetic inferences from the past were then linked to a 15,000-year record of spruce pollen accumulation rates (PAR) obtained from the same sediment core and used to infer past population size changes. PAR values represent the number of pollen grains accumulated at the sediment surface (cm^2^) in a year and they can be directly related to the population size of the studied taxon in the effective pollen source area of the examined lake [31]. The importance of this relationship has been long recognized by palaeoecologists [32] and used to reconstruct past population growth rates [33,34]. Recently, the collection of forestry data allowed direct comparison of surface sediment PAR values with actual biomass and population sizes in modern forests and thus the projection of PAR-based population size estimates back in time [35].

## Results

We analyzed eight different cpDNA regions in extant and fossil material from Norway spruce and six of them showed polymorphism (Table 1). The alignment of the extant sequences revealed 13 point mutations and five length polymorphisms that yielded nine haplotypes (Table 1 and Additional File 1). Two haplotypes were the most common (Ht2 and Ht8) found in 15 and 22 out of 58 individuals, respectively. Out of 462 fossil pollen grains analyzed we obtained totally 28 positive amplifications (three amplifications for fragment B, four for fragment D, ten for fragment TL, seven for fragment LF, two for the non-variable fragment CK, and finally two for the non-variable fragment MD). The higher amplification success (16.5%) obtained with pollen compared to Parducci *et al. *[7] was likely due to the choice of using more (usually five) pollen grains instead of one during amplification trials, or it may simply indicates favorable condition for DNA survival in the lake sediment at the Retezat. After sequencing, four of the 28 amplicons (two obtained with primers B and two with primers D) showed to be of *Pinus *origin, while seven amplicons obtained with primers LF were of *Abies *origin. Pollen grains of these two taxa were present in the examined sediment layer [30] and they were likely selected by mistake during the analyses. The rest of the amplicons were assigned to *P. abies*. Another possible explanation for the findings of *Pinus *and *Abies *sequences is DNA contamination from molecules of the surrounding sediment that may permeate or adhere to macrofossils in the sediments [36]. Nevertheless, these sequences still provide authentic early Holocene DNA information from *Pinus *and *Abies *individuals.

**Table 1:** Base substitutions detected in extant and ancient cpDNA sequences of Norway spruce (*Picea abies*)

cpDNA region	CK	MD	B		D			TL				Li		LF		K2i		
		
Variable position			88	107-126	77	78	82	168	196	197	206	154	157	115	133	114		
Position total			424	443 - 462	629	630	634	834	872	873	882	1081	1084	1279	1297	1464	Seq. length	Rel. freq.
Extant haplotypes																		
Ht1.	√	√	C	12A + 5G	A	A	C	G	T	C	G	T	C	T	T	C	1551	0.103
Ht2.	√	√	C	11A + 6G	A	A	C	G	T	C	G	T	C	A	T	C	1551	0.259
Ht3.	√	√	C	11A + 6G	C	A	C	G	T	C	G	T	C	A	T	C	1551	0.103
Ht4.	√	√	C	10A + 6G	A	A	C	G	T	C	G	T	A	A	T	T	1550	0.017
Ht5.	√	√	C	13A + 5G	A	A	C	G	T	C	G	T	C	A	T	C	1552	0.034
Ht6.	√	√	C	12A + 5G	C	A	C	G	T	C	G	T	C	A	T	C	1551	0.034
Ht7.	√	√	C	10A + 6G	A	A	C	G	T	C	G	T	C	A	T	C	1550	0.017
Ht8.	√	√	C	12A + 5G	A	A	C	G	T	C	G	T	C	A	T	C	1551	0.379
Ht9.	√	√	T	12A + 4G + 3A + G	A	C	T	G	T	A	G	C	C	A	G	C	1554	0.017
Macrofossil haplotypes																		
Cone	√				C	A	C	G	T	C	G			A	T	C	901	
S 3.					A	A	C										124	
S 4.												T	A				237	
S 8.		√			C	A	C	G	T	C	G						575	
S 9.	√		C	**10A + 7G**A + 7G				G	T	C	G			A	T	C	990	
S 10.		√			A	A	C	G	T	A	G	T	C			C	1016	
S 502-S 545			C	12A + 5G													211	
Fossil pollen haplotypes																		
P1.			C	10A + 6G													212	
P2-P3.					C	A	C										124	
P4-P11.								G	T	C	G						251	
P12.								G	T	A	G						251	
P13.								G	**G**	**A**	G						251	
P14-P15.	√																136	
P16-P17.		√															200	

(√) successful amplification of the fragment with no variation in the sequence; length of concatenated sequences is shown on the right; rows below the name of the fragments indicate the nucleotide position in the single fragments and in the concatenated sequence, respectively; base substitutions in bold were found only in the fossil material; Ht: haplotype; S: seed; P: pollen

Out of the investigated six Norway spruce seeds, DNA was successfully amplified from five seeds. Altogether, we obtained 15 positive amplifications (one for fragment B, three for fragment D, three for fragment TL, two for region Li, one for region LF, two for fragment K2i, one for the non-variable CK fragment, and two for the non-variable fragment M) (Table 1). When sequences were concatenated, the total length of the haplotypes varied between 124 and 1016 bp in the five seeds, and between 136 and 251 bp in pollen. Concatenation of the four regions amplified from the 4 cone scales gave a haplotype with a total length of 901 bp. Because cpDNA is paternally inherited in Norway spruce, sequence difference between cone and seed haplotypes (mother-tree *versus *offspring) were not unexpected. We found three transversion substitutions at position 77 in fragment D (C > A), position 197 in fragment TL (C > A) and position 157 in fragment Li (A > C). Finally, replication of the analyses done at Uppsala University using four new seeds retrieved at 502, 513, 525 and 545 cm depths in the same sediment, gave two positive amplifications (from sample 502 and 545 cm) using primers B. Both sequences were of Norway spruce origin and showed the 12A + 5G block, the most common variant present in the extant population.

### Comparison of ancient and extant sequences

After manually editing for base call errors, we performed an alignment between homologous regions obtained in extant and ancient individuals. The alignment revealed mismatches at several positions. Transition substitutions (G > A and C > T) were interpreted as incorrect nucleotide PCR incorporations due to post-mortem lesions in the ancient DNA molecules, a common problem when amplifications are performed from degraded DNA molecules [5]. We therefore neglected two G > A substitutions detected in sample P13 (fragment TL, positions 168 & 206 in Table 1). The rest of the substitutions were considered authentic, i.e. due to true polymorphism. Two variants were unique to the ancient population: a 10A + 7G repetition found in sample S9 (fragment B), and a GA dinucleotide substitution present in sample P13 (fragment TL). As expected, variants that were common to the extant population were also found in the ancient material (e.g. the common A, A, and C nucleotides at positions 77, 78 and 82 in fragment D was found in seeds S3 and S10). However, also rare variants found in extant individuals were detected among the ancient samples. For example, the 10A + 6G repetition in fragment B, and the C > A substitution at position 157 in fragment Li, both with a frequency 0.017 in the extant population, were found in sample P1 and S4, respectively. Similarly, a C > A substitution at position 197 in fragment TL was detected in samples P12 and S10.

### Population genetic analyses

Estimates of nucleotide diversity, haplotype diversity and average number of nucleotide differences calculated at the six polymorphic cpDNA loci are presented in Table 2. Observed values for fragment D, TL and Li suggest that the ancient spruce population harbored higher levels of genetic variation compared to the extant population. F_st _values at these loci were also statistically significant, an unusual result considering the low level of differentiation usually observed among populations of conifer species. Nucleotide-based genetic differentiation (K_s _and K_st_,) was also statistically significant at these three loci. On the whole, our results suggest differentiation between extant and ancient spruce populations that inhabited the areas surrounding Tăul dintre Brazi at 10,700-11,000 cal yr BP.

**Table 2:** Genetic diversity and population differentiation estimates in ancient and extant Norway spruce (*Picea abies*) populations based on six cpDNA loci

		**No. haplo-types**	**Haplotype diversity (Hd)**	**Nucleotide diversity (π)**	**Average no. differences (k)**	**Fst**	**Ks**	**Kst**	
	
B	Ancient	3	0.8333	0.0038	2.6667	0.0000	**0.9208**	**0.0000**	ns
	Extant	5	0.5777	0.0005	0.8004				
	
D	Ancient	2	0.5333	0.5333	0.5333	0.3266	**0.3592**	**0.0937**	**
	Extant	3	0.3001	0.3410	0.3409				
	
TL	Ancient	3	0.3846	0.0026	0.7912	0.0580	**0.1842**	**0.4248**	*
	Extant	2	0.0351	0.0001	0.0351				
	
Li	Ancient	2	0.6667	0.0028	0.6667	0.4569	**0.1**	**0.2304**	**
	Extant	3	0.0696	0.0003	0.0702				
	
LF	Ancient	1	0.0000	0.0000	0.0000	0.0938	0.2458	0.0036	ns
	Extant	3	0.2500	0.0014	0.2544				
	
K2i	Ancient	1	0.0000	0.0000	0.0000	0.0000	0.0333	0.0000	ns
	Extant	2	0.0351	0.0002	0.0351				

** significant at p < 0.01; * significant at p < 0.05; ns - not significant

### Phylogenetic relationships of ancient and extant specimens

**Figure 3:**
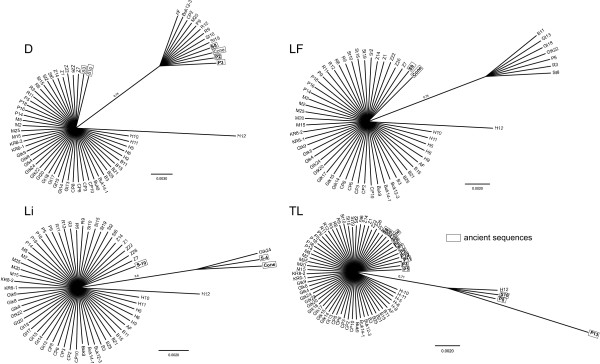
**Phylogenetic tree based on B/MCMC analysis of D, LF, Li and TL chloroplast fragments with ancient sequences indicated in boxes**. Posterior probabilities are shown along the branches.

Results of the phylogenetic analyses conducted on separate loci based on all sequences available (data set c in the Methods) for fragments D, TL, Li and LF are presented in Figure 3. A small group of sequences is formed by nine extant and four ancient sequences in the case of fragment D, while the other two ancient sequences are grouped with the rest of the extant material. In the case of LF fragment, the ancient sequences obtained from the cone and seed S8 are clustering together with the majority of the extant samples, while a smaller group of extant sequences that included samples collected on the northern slopes of the Retezat, form a well-supported cluster. The phylogenetic analysis conducted on fragment Li grouped together the majority of extant samples with one ancient sequence obtained from seed S10, while the other two ancient sequences form a well-defined clade with one extant sequence collected on the shores of Tăul dintre Brazi. For fragment TL we obtained the largest number of sequences from the ancient material and the majority of them grouped together with the extant specimens. Only three ancient and one herbarium specimen form a well-separated group.

**Figure 4:**
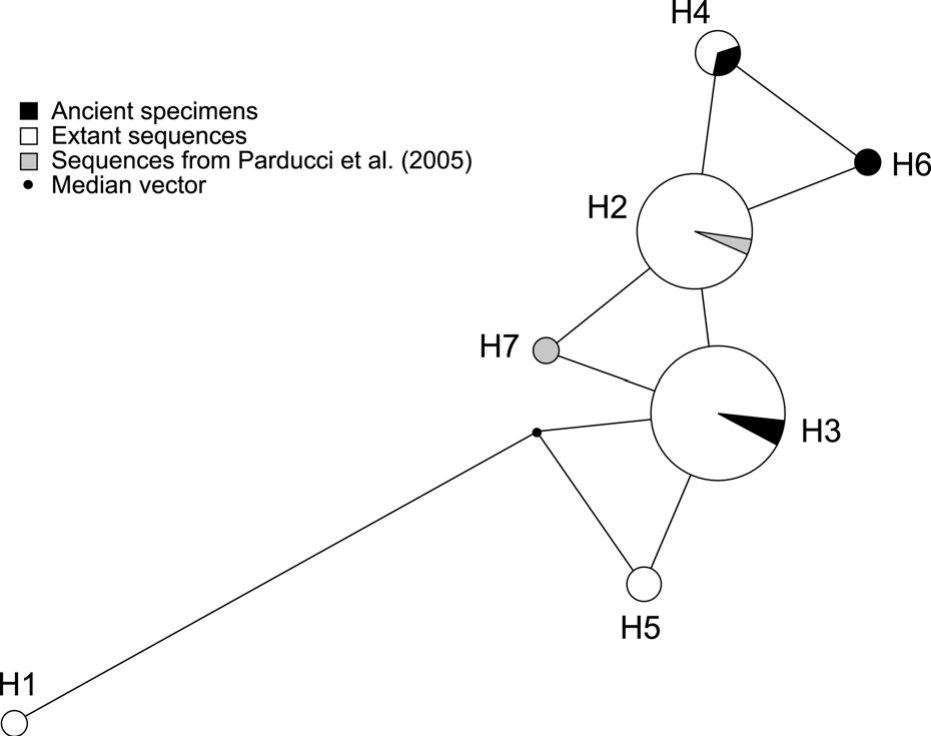
**Median-joining haplotype network of the chloroplast fragment B**. Numbers denote haplotypes found for fragment B only. Haplotype 1 corresponds to Ht9 (Table 1); haplotype 2 includes Ht2, Ht3; haplotype 3 includes Ht1, Ht6, Ht8; haplotype 4 includes Ht 4, Ht7 and P1; haplotype 5 corresponds to Ht5; haplotype 6 corresponds to S9; haplotype 7 corresponds to haplotype R in Parducci *et al. *(2005). For information on the ancient and extant haplotypes see Table 1 and Additional File 1.

Finally, the a median-joining network created using microsatellite B sequences (Figure 4) grouped the two previously identified 100-year-old spruce pollen from Sweden [7] together with the common haplotype detected in this study in extant and herbarium specimens. Our ancient sequences were also similar to this common extant haplotype with a single mutation step away from it, thus suggesting genetic continuity between Norway spruce populations.

### PAR-based population size estimates

**Figure 5:**
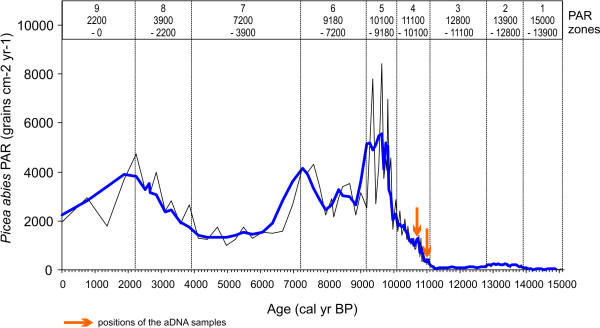
**Lateglacial and Holocene pollen accumulation rates (PAR) of Norway spruce (*Picea abies*) from Tăul dintre Brazi in the Retezat Mountains**. The dashed line on top of the PAR curve displays a three-term running mean.

**Figure 6:**
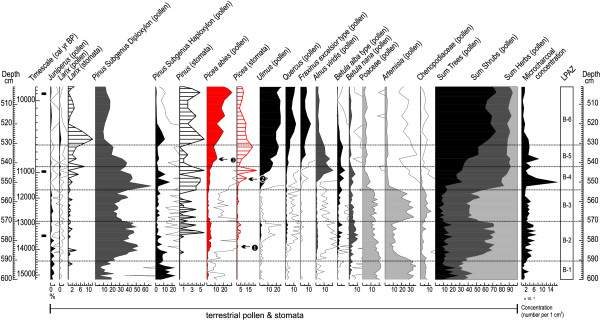
**Pollen and stomata record of Tăul dintre Brazi plotted against depth and calibrated BP timescales**. Norway spruce (*Picea abies*) fossils are highlighted in red. LPAZ: local pollen assemblage zones as in Table 3; ➊ first occurrence of spruce stomata at 13,900 cal yr BP; ➋ -➌ early Holocene population expansion of spruce between 11,750 - 10,780 cal yr BP inferred by pollen and stomata increases. Black rectangles along the timescale bar indicate the position of the radiocarbon dated samples.

Figure 5 shows pollen accumulation rates (PAR) of Norway spruce calculated at Tăul dintre Brazi for the last *ca*. 15,700 cal yr BP. PAR values are very low until 11,100 cal yr BP (0-270 grains cm^-2^yr^-1^) with an average of 120 grains cm^-2^yr^-1 ^that indicate very small population size in the Retezat Mts (*ca*. 1.2 - 6.4 trees per hectare according to the equation of Seppä *et al. *[35] for the Finnish woodlands). The Lateglacial interstadial, between 14,450 and 12,900 cal yr BP (zone 2 in Figure 5) show moderately higher PAR values (av. 155 grains cm^-2^yr^-1^). These values, together with the concurrent findings of spruce stomata (Figure 6), suggest the presence of a small lakeshore population during the Lateglacial interstadial at the study site. It is likely that spruce was absent from the lakeshore prior to this period and for a short period during the successive Younger Dryas climatic reversal (zone 3), between *ca*. 12,600-12,400 cal yr BP. From *ca*. 11,100 cal yr BP (period 4 and 5) a massive increase in spruce PAR, stomata concentration and pollen frequencies commenced with PAR values increasing from 200 to nearly 7000 grains cm^-2^yr^-1 ^in about 1000 years. Such values are equivalent to an approximate population size increase from 9 to 101 trees per hectare [35]. It should be noticed however, that due to approximation in the PAR-based population size extrapolations only the magnitude of this change is certain. Subsequently, the highest overall Holocene PAR values and hence inferred population size was detected between *ca*. 10,100 and 9180 cal yr BP (av. 4886 grains cm^-2^yr^-1^) followed by a modest decline, with still high PAR values until 7200 cal yr BP (zone 6). At this time, a significant decrease suggests also a decrease in population size (from *ca*. 252 to 120 trees per hectare on the basis of Seppä *et al. *[35]). These values were maintained until *ca*. 3900 cal yr BP and were followed by a second significant increase between 3900 and 2200 cal yr BP (zone 8). Finally, the PAR curve suggests a third decrease in population size after 2200 cal yr BP. This period coincides with the late Iron Age increase in livestock and the development of high altitude seasonal pasturing in the Romanian Carpathians [37] suggesting intensive timber exploitation by humans in the region. Overall, the PAR record suggests that following an early Holocene massive population increase on the northern slopes of the Retezat, the spruce population size did not remain constant, but showed marked fluctuation with three clear population bottlenecks dated to *ca*. 9180, 7200 and 2200 cal yr BP.

## Discussion

Patterns of plant genetic diversity in Europe show that levels of diversity usually decrease during postglacial colonization from the refugia [25,38-41]. There can be however large differences between colonization routes followed by different plant species due to climatic and environmental factors, altitudinal range expansion and contraction, proximity to the ice sheets, and topography. Results from our study suggest that these factors were crucial in determining the distribution and demography that shaped the genetic structure of the populations of the South Carpathians in the Retezat. In Central Europe, high levels of diversity are usually found in the oldest spruce regions ( i.e. where spruce pollen is present since at least the Early Holocene), in the south-eastern Alps, southern Bohemian Massif, West Carpathians and northern Dinaric Alps [25]. The only exception is the South Carpathian refugium, where scientists have found unique haplotypes and relatively low diversity levels in extant populations [25]. Indeed, in our study we found a decreased genetic variation in the extant populations in the Retezat compared to the ancient population, and such lower diversity could be easily associated with the several population bottlenecks identified by the PAR data. Nevertheless, we cannot exclude that the limited number of fossil sequences affected the interpretation of the aDNA data.

It is reasonable to assume that the fluctuations observed in population size after an early Holocene rapid expansion at Tăul dintre Brazi had a substantial impact on the genetic structure of the spruce population. The pollen record shows that a small population was already established in the lateglacial period around 13,900 cal yr BP at high altitudes formerly covered by glacier ice (Figure 5 and 6) supporting the presence of a refugium in this area. Such a refugium has been also detected by genetic analysis conducted on living populations by Bucci & Vendramin [22] and Tollefsrud *et al. *[25]. Assuming therefore that spruce was present and probably abundant throughout the last glacial cycle at lower elevations in the Retezat, it is likely that our sampled ancient population is similar in genetic composition to this refugial population. The pollen and the stomata record (Figure 6) show that the lateglacial expansion came to a halt during the Younger Dryas climatic reversal (between *ca*. 12,900 and 11,500 cal yr BP), and the spruce population was greatly reduced and/or eradicated from the lakeshore [30]. The species expanded again during the early Holocene and our ancient material was sampled at the beginning of this second population expansion that started around 11,100 cal yr BP. Such increase in population size started from a population that was very small, according to the PAR record about 1/10 or 1/5 of the extant one, and that successively increased between *ca*. 11,100 and 10,100 cal yr BP. A significant increase in population size, however, only began around 10,000 cal yr BP, when the largest overall Holocene distribution was reached. Fossil pollen data from this site (Figure 5) indicates that between 10,100 and 9180 cal yr BP Norway spruce replaced European larch (*Larix decidua*) on the lakeshore and probably also in other areas at the same altitudinal belt [30]. By this time, the population increased to a number that likely exceeded the extant population size.

According to the PAR record, the maximum extension of Norway spruce in the Retezat was followed by three distinct population bottlenecks. The first reduction occurred at 9180 cal yr BP and was likely caused by a local population decline due to the expanding lake surface area [42]. A second reduction in size occurred at 7200 cal yr BP and it was attributable to the climate-induced spread of European hornbeam (*Carpinus betulus*) that partially replaced former mixed Norway spruce - hazel forests at mid elevations [27,42]. The third bottleneck started 2200 years ago and was of anthropogenic origin as suggested by the accompanying decrease in many other arboreal elements and the spread of human-indicator herbs [42].

Typically, population bottlenecks are characterized by loss in allelic richness and a more limited decrease of genetic diversity at neutral loci [43]. This theory however is not strictly applicable to the events that seem to have occurred during postglacial recolonization in the Retezat. In our case, a combination of factors may have acted to limit the initial loss in allelic richness (original large population sizes in the early Holocene), and later to increase it (population bottlenecks and range fragmentation due to forest management). Several pollen studies and historical documents show indeed that forest management was intense from 2200 cal years BP and destroyed the large continuous distribution of Norway spruce in this region [27,44,45].

Results from the genetic and PAR analyses seem therefore to support the hypothesis that the higher ancient genetic variability and the absence of some ancient haplotypes in the extant spruce populations was due to repeated bottlenecks experienced during the Holocene in the Retezat. In addition, as suggested by Tollefsrud *et al. *[25], the dry plains of central and south-eastern Europe, may also have acted as an ecological barrier to the expansion of the early Holocene populations from this site, whereas humid mountain ranges elsewhere in central Europe facilitated the northward spread of new spruce lineages in the successive periods. As a result, despite the proximity to the South Carpathian refugium, the genetic diversity of this site declined during the Holocene.

## Conclusions

This study demonstrates how past population size changes inferred from PAR records can be efficiently used in combination with aDNA data. The joint application of palaeoecological and population genetic analyses proved to be a powerful tool to understand the influence of past population demographic changes on the haplotype diversity and genetic composition of an important forest tree species. We used a pollen and plant macrofossil-based aDNA approach and traced population size changes of Norway spruce in a refugial area in the South Carpathian Mountains. Results clearly show that the paternal lineage observed at the beginning of the Holocene in the Retezat Mts persisted at the site until today. Problems associated with low sample size in our aDNA data could be overcome by the information obtained from the PAR data that clearly showed significant population size fluctuations in the Retezat Mts during the Holocene. This combined information support the hypothesis that the spruce population of the Retezat Mts underwent significant demographic changes, most probably due to climatic shifts and anthropogenic influences, and that likely these changes significantly altered the haplotype frequencies and the level of genetic diversity.

This study thus laid down the first basis for further aDNA analyses. By examining additional levels of this sediment profile we can provide further insight to support the hypotheses presented in this study.

We have shown also that longer sequences, and more robust phylogenetic inferences can be obtained from plant macrofossils like seeds and cone scales when aliquots of the DNA extracts are used in multiple PCR reactions providing sequences from different cpDNA regions. Similar attempts performed with fossil pollen grains were not as successful limiting at the moment population genetic inferences to single loci. On the other hand, multiple PCR products (obtained by applying multiple primer sets in one reaction) and thus longer sequences might be obtained from pollen using the new generation sequencing platforms, like the 454 GS FLX [14,46] or multiple-loci PCR analyses. With this type of analyses, the use of DNA extracted from plant macrofossils allows the study of longer DNA sequences or, in the case of the 454 platform, even complete genomes from ancient plant specimens providing good resolution data sets for population genetic analyses, as recently done with an extinct cave bear species [47]. The main obstacle to the use of plant macrofossils for detecting population level changes is the limited number of individuals that can be obtained from a sediment sample. In cases, however, where macrofossils are well preserved and retrieved in large quantities from multiple time horizons [48], they can easily provide multiple genotypes suitable for population genetic studies similarly to our recovery of an intact 11,000-year-old Norway spruce cone from Tăul dintre Brazi. In such cases, the opportunity offered by new data analytical methods in population genetics that work with multi-locus data, enables an efficient use of a relatively small number of individuals in the reconstruction of past population demographic changes [49]. Indeed, there are several fossil sites in Europe where Upper Weichselian (ca. 11,500 - 45,000 cal yr BP) plant macrofossils from single or multiple chronological horizons have been recovered in large numbers [24]. In the Carpathians, for example, numerous *Picea *sp., *Pinus cembra *and *Larix *sp. cones have been reported from several last glacial (15,000-45,000 cal yr BP) and Holocene peat sediments [50,51]. Such material seems ideal for aDNA analysis, as both organellar and nuclear DNA in the seeds of these cones hold many alleles. Its study have the potential of revealing ancient genetic diversity in cryptic northern refugia and adding valuable information on the role of this area in the postglacial recolonization of northern and western Europe [11].

Our analyses in particular proved that the use of aDNA in combination with PAR data obtained from the same site can be very efficient in discovering demographic changes that occurred in the past in Norway spruce, and will hopefully be used in other wind-pollinated tree species. Finally, PAR-inferred demographic scenarios offer the opportunity of being incorporated in coalescence-based Bayesian mutation rate estimations, and this way more accurate substitution rates can be obtained [52].

## Methods

### Ancient material

Our ancient material came from the early Holocene part of a 5-m long sediment sequence extracted at Tăul dintre Brazi (0.5 ha, 1740 m a.s.l.). The lake is situated in a north-facing slope of the Retezat Mts, South Carpathians (Figure 1) and was formed in a glacial basin approximately 15,700 years ago, directly after the retreat of the Lolaia glacier from this valley [42,53]. Today, the forest around the lake consists of *P. abies, Pinus cembra *(Stone pine)*, Pinus mugo *(Mountain pine) and *Rhododendron myrtifolium*. The core was analysed for fossil pollen, plant macrofossils, and an age-depth model was constructed based on eleven radiocarbon determinations of terrestrial plant macro remains (Additional File 2) [42]. Pollen preservation was excellent throughout the entire core. The sediment sample from which the spruce pollen grains were retrieved for DNA analysis was organic-rich gyttja showing the first major concentration increase of spruce pollen (533 cm; Figure 2). The age of the studied layer was 10,700 cal yr BP.

An intact Norway spruce cone at 545 cm sediment depth and dated 11,000 cal yr BP [42], was used for aDNA analysis (Figure 2). Half of this cone was sent to the Poznań Radiocarbon Laboratory for dating, while the other half including 6 seeds and 4 cone scales was used for aDNA analysis. The fossil spruce pollen and cone were analyzed in the arche DNA laboratory of the Hungarian Natural History Museum (NHMUS).

Replication of the aDNA analyses was performed at Uppsala University, where DNA from four additional seeds were analyzed. These seeds were retrieved from the same sediment at 502, 513, 525 and 545 cm depths corresponding to 9972, 10,145, 10,331 and 11,000 cal yr BP, respectively.

### Pre-PCR preparation of the fossil samples - Molecular Taxonomy Laboratory at the Natural History Museum of Budapest, Hungary (NHMUS)

In a clean-air, DNA-free room (room 1) at the archeDNA laboratory of *NHMUS*, one cm^3 ^sediment was removed from the inner section of the core at 533 cm using sterile scalpels (Figure 2). The sample was sieved through 120 μm mesh and stored in sterile distilled water at 2°C until further treatment. Aliquots of the filtrate were examined under a light microscope at magnification x100 (Olympus CX 42). Spruce pollen grains were isolated and serially moved between water drops on microscope slides using sterile Hamilton syringes. Totally, we selected 462 fossil pollen grains from sediments dated 10,700 cal BP and stored them at -80°C until aDNA analysis. For PCR set up we followed the methods described in Parducci *et al. *[7], but we increased the number of pollen grains up to five in each aliquots prepared for amplification and used a finer mesh (120 μm instead of 180 μm) to obtain more efficient concentration of the grains.

Seeds and cone scales were separated from the 11,000-year-old fossil cone under a sterile hood irradiated by high intensity UV light (1 J/cm^2^, 1 hour, several times, turning around the particles at each step to decontaminate the whole surface). All lab ware used for manipulations (forceps, tubes, pestles and mortars, etc.) was nightly UV-irradiated.

In a second room in the arche DNA lab (room 2) we transferred the pollen grains on sterilized microscope slides using a sterile Hamilton syringe and washed them separately ten times with UV-irradiated sterile water. After washing, we moved five to six grains to separate UV-irradiated sterile PCR microtube with 5 μL UV-irradiated sterile water. Along with each tube, we prepared also a negative control including 5 μL of the last drop of water used for washing the five grains. The tubes were stored at -20°C and before PCR they were transferred to the third room in the arche DNA lab (room 3) where the frozen pollen grains were crushed on ice with a sterile pestle in 12 μL sterile distilled water. Five μL aliquots were immediately used as template for PCR. Each pollen sample was amplified with one primer pair. A reagent blank and a negative control including 5 μL of the last washing water were prepared for each amplification.

DNA from seeds and cone scales was extracted in room 3. We manually powdered the material using a NucleoSpin Plant II Kit (Macherey-Nagel) and following the CTAB protocol provided by the manufacturer in 100 μL elution volume. Aliquots of each seed DNA extract was amplified with all primers used in this study.

### DNA amplification and sequencing

We selected eight primer pairs (six specifically designed for this study) that amplified short and variable and neutral regions from the spruce cpDNA (pseudogenes, microsatellites, intergenic spacers and introns). In addition, we used two cpDNA microsatellite primer pairs previously used by Parducci *et al. *[7]. The name of the amplified regions, the sequence of the primers and the expected length in Norway spruce of the eight fragments are listed in Table 3. The primers developed for this study were designed using Clone Manager Suite 7 (SciEd) using *Pinus thunbergii *and *P. sitchensis *reference sequences and *P. abies *chloroplast cpDNA sequences retrieved from GeneBank.

**Table 3:** Targeted regions, primer sequences and approximate product length (bp) of PCR for primer pairs used to amplify six cpDNA regions of Norway spruce

Region (short name)	cpDNA region	Primer	Sequence (5' - 3')	Position in cpDNA of *Pinus thunbergii *NC_001631	Position in cpDNA of *Picea sitchensis *NC_011152	Fragment Length in *P. abies *(bp)
ndhCp-ndhKp (CK)	pseudogene	ndhK-for1	CACTTCAGTTCTTGTTGTTCC	66530→66550	66774→66794	136 bp
		ndhC_ndhK-rev	TCGCCACAGAACCAACGATG	66666→66647	66910→66891	
psbM-trnD (MD)	intergene spacer	psbM_trnD-f1	GTTCGAGTAACGGAATCTAAC	28004→28024	27925→27945	200 bp
		psbM_trnD-rev1	CGAACGTCTTCTGGAGTAGC	28204→28185	28124→28105	
Pt3024, ORF46b* (B)	microsatellite	B-for	GCTTATGGCATTGTTGATGT	30166→30185	30701→30720	212-216 bp
		B-rev	TGGGCATTCTAGCTGTATTG	30387→30368	30941→30922	
Pt15169, ORF84* (D)	microsatellite	D-for	CTTGGATGGAATAGCAGCC	15169→15187	15208→15226	124 bp
		D-rev	GGAAGCGCATTAAGGTCATTA	15286→15266	15327→15307	
trnT-trnL (TL)	intergene spacer	trnT_trnL-sp-f1	CTGAGCTAAGCAGGCTCAATGG	69189→69168	69573→69552	251 bp
		trnT_trnL-sp-brev	GCATGTTATTATCCTCCCCTAG	68945→68967	69323→69344	
trnL (Li)	Intron	trnL-intron-f2	GAACGCTCTATTTACACC	68497→68480	68884→68867	237 bp
		trnL-intron-rev	ACACGTAGAATTGGACTCTATC	68261→68282	68648→68669	
trnL-trnF (LF)	Intergene spacer	Aa_trnLF-for	GGTTCAAGTCCCTCTATCCC	68198→68179	?	186 bp
		Aa_trnLF-rev	ACTGATCAACTCAACTTGTCATAAGATGG	68014→68042	68400→68428	
matK-trnK (K2i)	intron	trnK2i-f1	GCCCTCGTTCATGAGAATAACC	3281→3302	3368→3389	204 bp
		trnK2i-r1	CATGAGTCAGGAGAGCGATTGG	3477→3456	3573→3552	

* cpDNA region names from Parducci *et al. *(2005); primers B and D are from Parducci *et al. *(2005); primers CK, MD, TL, Li, LF and K2i were designed specifically for this study

DNA amplifications were performed on all DNA extracts (from pollen, seeds and cone) using single primers pairs. We used 20 μL that included 5 μL of template solution (regardless of the source), 1x reaction buffer, 1.5 mM MgCl_2_, 0.1 mM dNTP (each), 0.25 mM forward and reverse primers, and 0.4 U of AmpliTaq Gold polymerase (Applied Biosystems). The PCR profile was 10 min at 94°C, 45 cycles of 20 sec at 94°C, 30 sec at 55°C, 1 min at 72°C and final elongation 7 min at 72°C. Five μL of the PCR reactions were tested for amplicons on 2% agarose by gel-electrophoresis in 0.5x TBE and stained with ethidium bromide. In case of successful amplification, 2-3 μL of the first PCR product was reamplified and purified using High Pure PCR Product Purification Kit (Roche) in a PCR room in the modern DNA lab at NHMUS where spruce had never been processed before (room 4). Finally, DNA sequencing reactions were prepared in a fifth room at *NHMUS *(room 5) using 10 ng of purified PCR products using forward and reverse primers and BigDye Terminator v1.1 Cycle Sequencing Kit (Applied Biosystems). After purification the products were run on a 3130 Genetic Analyzer (Applied Biosystems).

### Analysis on extant material

Fresh needles were collected from 52 adult trees of extant spruce populations located in the Retezat Mts. In addition, we sampled needles from 6 herbarium specimens (5 from the Carpathians, 1 from the Austrian Alps) (Figure 1; Additional File 3). Needles were stored at 4°C until use, and total genomic DNA was extracted with DNeasy Plant Mini Kit (QIAGEN). PCR amplifications and DNA sequencing were performed after all fossil DNA analyses had been completed in room 6 that was physically separated from rooms 1-3 at the archeDNA laboratory, and also from room 4 in the modern DNA laboratory, using the same procedures as for the fossil material.

Altogether 833 sample and 322 control PCR reactions were done on the modern and extant material at *NHMUS *(without re-amplification and sequencing PCRs).

### DNA amplification, cloning and sequencing - Molecular Genetic Laboratory (EBC, Uppsala University, Sweden)

Replication of the aDNA analyses was performed on four seeds using the Quiagen Multiplex PCR kit and increasing the number of amplification cycles to 38. This kit contains a built-in hot start polymerase enzyme and a unique PCR buffer that makes it appropriate for the amplification of difficult templates like in the case of fragmented aDNA. We run single reactions with single primer pairs at a time in 20 μL volume, including 3 μL of extracted DNA solution, 1X Multiplex PCR Master Mix (QIAGEN), 0.2 μM of each primer, and water for adjusting the final volume. After amplification 5 uL of PCR products visible as bands of the expected size on 2% agarose gel were purified using ExoSAP-IT (Affymetrix, Inc.). Two μL of purified product was used directly for cloning using the CloneJet PCR Cloning Kit (Fermentas). Two μL of the purified products were ligated into pJET1.2/blunt Cloning Vector in the presence of T4 DNA ligase at room temperature for 30'. Fifty μL of competent DH5 cells were transformed using 5 μl of the ligation and grown in SOC solution for 90' at 37°C. The cells were plated on LB-ampicillin plates. Colonies were screened in 10 uL PCRs using pJET universal primers and following manufacture's instructions. All clones with inserts of expected sizes were sequenced using Macrogen DNA Sequencing service in Korea.

### Precautions against contamination and criteria used for sequence authentication

We extracted DNA in environments specifically dedicated to aDNA studies (see methods). At *NHMUS *in Hungary and at *EBC *in Sweden all analyses on ancient material were performed in separate DNA-free rooms, in laboratories specifically dedicated to aDNA work and physically separated from laboratories where the modern DNA analyses were performed. Analyses on extant material were performed after aDNA analyses had been completed. No amplifications were obtained in extraction and reagent controls and amplifications from aDNA extracts always produced PCR products visible on agarose gel as single and clear bands.

Cloning is generally performed in the case of aDNA studies to detect mosaic sequences as a result of jumping PCR between sequences, to identify amplification errors due to post-mortem DNA damage in the template and to detect PCR-slippage in the case of microsatellites [20,54]. Initially we planned to sequence PCR products using forward and reverse primers to detect sequence variants after cloning. Because we found no sequence variation in our products after three independent cycle-sequencing assays, we disregarded the cloning step at *NHMUS*. The multiple cycle-sequencing method also allowed us to exclude the presence of artifacts resulting from PCR-slippage in the microsatellite B region. Importantly, generation of mosaic sequences was excluded in our case due to the uniparental inheritance of the chloroplast genome in spruce.

### Data analysis

Ancient and extant DNA sequences were manually aligned in MEGA v4 [55] and identity was verified using BLASTN [56]. The 10,700-year-old DNA sequences obtained from pollen were analyzed together with the sequences derived from fossil seeds and cone scales at *NHMUS *and *EBC*.

To compare genetic diversity in ancient and extant spruce populations, we estimated haplotype (H_d_) and nucleotide diversities (π, [57]), average number of nucleotide differences (k, [58]), weighted average of the number of differences between sequences from two populations (K_S_, averaged with a weight of w = n_1_/(n_1_+n_2_), where n_1 _and n_2 _represent population sizes [59]), and an estimate of genetic differentiation between two populations (K_ST_, calculated from K_S _and the average number of differences between two sequences in the total sample [59]). We calculated the statistical support of K_S _and K_ST _values with permutation tests through 1000 randomizations [59]. Gene flow between extant and ancient populations was estimated using F_ST _[59]. K_ST _and F_ST _statistics are identical except for a different weighting with population sizes [59]. Alignment gaps were considered as fifth-state characters when calculating H_d_, k, K_S_, K_ST _and F_ST _values. We performed the calculations in DnaSP v 4 [60].

Phylogenetic relationships between ancient and extant sequences were analyzed using B/MCMC inference as implemented in MrBayes [61]. Only point mutations in the form of transversion were taken into consideration, disregarding also allele length polymorphisms of microsatellite B. In the phylogenetic analysis we also included two previously obtained 100-year-old spruce sequences obtained by Parducci *et al. *[7] using primer pair B. The nucleotide substitution model was selected using the Akaike Information Criterion in ModelTest 3.7 [62]. The analyses were run (a) on concatenated fragments using all ancient and extant sequences; (b) on concatenated fragments of all extant sequences and of the longest ancient sequences (obtained from cone scales and seeds S8, S9 and S10); (c) on single fragments on all sequences. Two parallel tests were run for 20 million generations in the case of dataset (a) and dataset (b) to achieve convergence. The determined substitution model was K81uf+I [63] in both cases, with a proportion *I *= 0.9667 of invariable sites for dataset (a) and *I *= 0.9606 for dataset (b). One cold and eleven heated chains were used in both analyses, sampling for every 10,000 generations. After discarding 25% of the samples as burnin, all identified compatible groups were added to consensus trees. The trees were visualized in FigTree [64].

Substitution model for dataset (c) were TrN for fragment B [55], F81 [65] for fragments D, LF, Li and TL and HKY for K2i [66], all without *Γ*-distributed or invariable loci. The parallel tests were run until achieving convergence (i.e. 5 million generations for fragment K2i, 16 million generations for Li, and 20 million for B, D, LF and TL). Sampling was performed in every 10,000 generations. Twenty-five percent of the samples were discarded as burnin and 50% majority rule consensus trees were constructed. The trees were visualized in FigTree.

To map haplotype relationships for fragment B, we also calculated a median-joining network [67] using the software Network v4.510 (http://www.fluxus-engineering.com/ accessed on September 25 2009). Two 100-year old Norway spruce pollen sequences from Parducci *et al. *[7] were included in this analysis, and the entire sequence set was truncated to the common length of 112 bp.

## Competing interests

The authors declare that they have no competing interests.

## Authors' contributions

ÁM, JN and EM designed the experiment. EM, MB and MB collected and sampled the extant material. EM provided the fossil samples and the palynological data information. IR conducted the taxonomic identification of the macrofossils. ÁM, JN, EM and LP conducted PCR and sequencing analyses. ÁM and MB performed sequence alignment and data analysis. EM, LP, MB and ÁM wrote the manuscript. All authors read and approved the final manuscript.

## Supplementary Material

Additional file 1 **Sequences of the cpDNA fragments in Norway spruce (*Picea abies*) with information on the region name, applied primer pairs, name of the fossil and extant samples and length of the sequence**. Bold and underlined bases refer to variable positions or blocks. Click here for file

Additional file 2 **Radiocarbon dates and age-depth modeling of sediment core TDB-1, Taŭl dintre Brazi (1740 m a.s.l), Retezat Mountains, South Carpathians, Romania**. Click here for file

Additional file 3 **Location and geographical coordinates of the extant Norway spruce (*Picea abies*) samples and herbarium specimens used for cpDNA analysis**. Click here for file
