# Supplementary material for: Population dynamics and genetic changes of Picea abies in the South Carpathians revealed by pollen and ancient DNA analyses
Source: BMC Evol Biol. 2011 Mar 10;11:66. doi: 10.1186/1471-2148-11-66 (PMC3068097; doi:10.1186/1471-2148-11-66)
Supplement: Additional file 1 — Sequences of the cpDNA fragments in Norway spruce (Picea abies) with information on the region name, applied primer pairs, name of the fossil and extant samples and length of the sequence. Bold and underlined bases refer to variable positions or blocks. [file 1471-2148-11-66-S1.DOC]

**Additional file 1 - Sequences of the cpDNA fragments in Norway spruce (*Picea abies*) with information on the region name, applied primer pairs, name of the fossil and extant samples and length of the sequence**

Bold and underlined bases refer to variable positions or blocks.

CK; ndhK-for1, ndhC_ndhK-rev; all samples (non-variable); 136 bases

CACTTCAGTTCTTGTTGTTCCTGATGTTGGAACAGTCTCCCTTTATCTATGGGCTATGAG

TTCTAGTATACAGGGTATATCTGTATTTATAAGAGCTTCAATTCTCGTGCTTATTTTCAT

CGTTGGTTCTGTGGCG

MD; psbM_trnD-f1, psbM_trnD-rev1; all samples (non-variable); 200 bases

GTTCGAGTAACGGAATCTAACTAACGGATTGAATGAATTCTTCGATGAAAATAGTATAAC

ATAATATGATCACTTTTGATAATACTAGTAAGAAAAACTTACGTGCATCAGAAAACGTTC

CGATCAACACATGTCTGTATCATTTCGAAAGAATAGGATTCGTACGAATAATAACCTTCT

GCTACTCCAGAAGACGTTCG

B (Pt3024); B-for, B-rev; C+12A+5G (haplotype 1, 6, 8); 213 bases

GCTTATGGCATTGTTGATGTCGTAGCGGAAGGTTGATCTCATATCGGAAGATCCTCTTTT

TAATTGATTTTTATAATGAACTGTAAA**C**TGATCTCTTCTTTTTCTT**AAAAAAAAAAAAGG**

**GGG**AAAGGGAAAGTGATTCATTTCATAATAACCTTATATGGTTAGGATCAATCCGAACCA

GTTGATTCCGCATACAATACAGCTAGAATGCCC

B (Pt3024); B-for, B-rev; C+11A+6G (haplotype 2,3); 213 bases

GCTTATGGCATTGTTGATGTCGTAGCGGAAGGTTGATCTCATATCGGAAGATCCTCTTTT

TAATTGATTTTTATAATGAACTGTAAA**C**TGATCTCTTCTTTTTCTT**AAAAAAAAAAAGGG**

**GGG**AAAGGGAAAGTGATTCATTTCATAATAACCTTATATGGTTAGGATCAATCCGAACCA

GTTGATTCCGCATACAATACAGCTAGAATGCCC

B (Pt3024); B-for, B-rev; C+10A+6G (haplotype 4,7 and P1) 212 bases

GCTTATGGCATTGTTGATGTCGTAGCGGAAGGTTGATCTCATATCGGAAGATCCTCTTTT

TAATTGATTTTTATAATGAACTGTAAA**C**TGATCTCTTCTTTTTCTT**AAAAAAAAAAGGGG**

**GG**AAAGGGAAAGTGATTCATTTCATAATAACCTTATATGGTTAGGATCAATCCGAACCAG

TTGATTCCGCATACAATACAGCTAGAATGCCC

B (Pt3024); B-for, B-rev; C+13A+5G (haplotype 5); 214 bases

GCTTATGGCATTGTTGATGTCGTAGCGGAAGGTTGATCTCATATCGGAAGATCCTCTTTT

TAATTGATTTTTATAATGAACTGTAAA**C**TGATCTCTTCTTTTTCTT**AAAAAAAAAAAAAGG**

**GGG**AAAGGGAAAGTGATTCATTTCATAATAACCTTATATGGTTAGGATCAATCCGAACCA

GTTGATTCCGCATACAATACAGCTAGAATGCCC

B (Pt3024); B-for, B-rev; T+12A+4G+3A+G (haplotype 9); 216 bases

GCTTATGGCATTGTTGATGTCGTAGCGGAAGGTTGATCTCATATCGGAAGATCCTCTTTT

TAATTGATTTTTATAATGAACTGTAAA**T**TGATCTCTTCTTTTTCTT**AAAAAAAAAAAAGG**

**GGAAAG**AAAGGGAAAGTGATTCATTTCATAATAACCTTATATGGTTAGGATCAATCCGAA

CCAGTTGATTCCGCATACAATACAGCTAGAATGCCC

B (Pt3024); B-for, B-rev; C+10A+7G (S9); 213 bases

GCTTATGGCATTGTTGATGTCGTAGCGGAAGGTTGATCTCATATCGGAAGATCCTCTTTT

TAATTGATTTTTATAATGAACTGTAAA**C**TGATCTCTTCTTTTTCTT**AAAAAAAAAAGGGG**

**GGG**AAAGGGAAAGTGATTCATTTCATAATAACCTTATATGGTTAGGATCAATCCGAACCA

GTTGATTCCGCATACAATACAGCTAGAATGCCC

D (Pt15169); D-for, D-rev; A+A+C (haplotype 1, 2, 4, 5, 7, 8 and S3, S10); 124 bases

CTTGGATGGAATAGCAGCCAACTCAGTAAATCTTCAGGTCCCTTTCTTTTTTCTATTTTC

TTATTATTACTAATTT**AA**TAT**C**TATTAGCCCTTCATATAGCTATAATGACCTTAATGCGC

TTCC

D (Pt15169); D-for, D-rev; C+A+C (haplotype 3, and cone, S8, P2, P3); 124 bases

CTTGGATGGAATAGCAGCCAACTCAGTAAATCTTCAGGTCCCTTTCTTTTTTCTATTTTC

TTATTATTACTAATTT**CA**TAT**C**TATTAGCCCTTCATATAGCTATAATGACCTTAATGCGC

TTCC

D (Pt15169); D-for, D-rev; A+C+T (haplotype 9); 124 bases

CTTGGATGGAATAGCAGCCAACTCAGTAAATCTTCAGGTCCCTTTCTTTTTTCTATTTTC

TTATTATTACTAATTT**AC**TAT**T**TATTAGCCCTTCATATAGCTATAATGACCTTAATGCGC

TTCC

TL; trnT_trnL-sp-f1, trnT_trnL-sp-brev; G+T+C+G (haplotypes 1-8, and cone, S8, S9, P4-P11); 251 bases

CTGAGCTAAGCAGGCTCAATGGAATATAACTCCTCATTTCATTGGTGTGAGATCCATAGA

TTCTTTTGGAATCCTAACGATTATAGCGCGAATCAGATTCAATGACGCAATCCAGATTAC

AATTACAACGGAACATTGTTTGAATGTAGATTGTAGATTCCTTCAAG**G**GAAAGAAAAGGG

AAGTAAGGATGAATT**TC**TATCGATC**G**TTTTACTCACTCTTCCAAATCGACTAGGGGAGGA

TAATAACATGC

TL; trnT_trnL-sp-f1, trnT_trnL-sp-brev; G+T+A+G (haplotype 9 and P12); 251 bases

CTGAGCTAAGCAGGCTCAATGGAATATAACTCCTCATTTCATTGGTGTGAGATCCATAGA

TTCTTTTGGAATCCTAACGATTATAGCGCGAATCAGATTCAATGACGCAATCCAGATTAC

AATTACAACGGAACATTGTTTGAATGTAGATTGTAGATTCCTTCAAG**G**GAAAGAAAAGGG

AAGTAAGGATGAATT**TA**TATCGATC**G**TTTTACTCACTCTTCCAAATCGACTAGGGGAGGA

TAATAACATGC

TL; trnT_trnL-sp-f1, trnT_trnL-sp-brev; A+G+A+A (P13); 251 bases

CTGAGCTAAGCAGGCTCAATGGAATATAACTCCTCATTTCATTGGTGTGAGATCCATAGA

TTCTTTTGGAATCCTAACGATTATAGCGCGAATCAGATTCAATGACGCAATCCAGATTAC

AATTACAACGGAACATTGTTTGAATGTAGATTGTAGATTCCTTCAAG**A**GAAAGAAAAGGG

AAGTAAGGATGAATT**GA**TATCGATC**A**TTTTACTCACTCTTCCAAATCGACTAGGGGAGGA

TAATAACATGC

Li; trnL-intron-f2, trnL-intron-rev; T+C (haplotypes 1-3, 5-8, and S10); 237 bases

GAACGCTCTATTTACACCTAAAAAGTGGGAATGTGATATAACATCAGACAAAACTCGCGA

TCAGAACTTGAATCGTTCCAAGCATCTATTCGTAAGATAGATGCCAGATTCGAGTTGAAG

TACTGATTTTACATTAAGTAATCCAATTATGAA**T**TT**C**TCTACTTTAGATAGAGAATTGAA

TCAGTTTTTGGAATAAATGGTTGGACGAGAATAAAGATAGAGTCCAATTCTACGTGT

Li; trnL-intron-f2, trnL-intron-rev; T+A (haplotype4 and S4); 237 bases

GAACGCTCTATTTACACCTAAAAAGTGGGAATGTGATATAACATCAGACAAAACTCGCGA

TCAGAACTTGAATCGTTCCAAGCATCTATTCGTAAGATAGATGCCAGATTCGAGTTGAAG

TACTGATTTTACATTAAGTAATCCAATTATGAA**T**TT**A**TCTACTTTAGATAGAGAATTGAA

TCAGTTTTTGGAATAAATGGTTGGACGAGAATAAAGATAGAGTCCAATTCTACGTGT

Li; trnL-intron-f2, trnL-intron-rev; C+C (haplotype 9); 237 bases

GAACGCTCTATTTACACCTAAAAAGTGGGAATGTGATATAACATCAGACAAAACTCGCGA

TCAGAACTTGAATCGTTCCAAGCATCTATTCGTAAGATAGATGCCAGATTCGAGTTGAAG

TACTGATTTTACATTAAGTAATCCAATTATGAA**C**TT**C**TCTACTTTAGATAGAGAATTGAA

TCAGTTTTTGGAATAAATGGTTGGACGAGAATAAAGATAGAGTCCAATTCTACGTGT

LF; Aa_trnLF-for, Aa_trnLF-rev; T+T (haplotype 1); 186 bases

GGTTCAAGTCCCTCTATCCCCACCTAGGTTCGTTCCCGAACGACTGATCTATTTTCTCCA

ATTCCATTAGTTCGAATCCATTCTCACTTCTCGATTATTTTACCTCACTATTTT**T**TTTCT

TCATGAACAGAA**T**AAATTAGAACATGAATCTGTCCATCCATCTTATGACAAGTTGAGTTG

ATCAGT

LF; Aa_trnLF-for, Aa_trnLF-rev; A+T (haplotype 2-8, and cone, S9); 186 bases

GGTTCAAGTCCCTCTATCCCCACCTAGGTTCGTTCCCGAACGACTGATCTATTTTCTCCA

ATTCCATTAGTTCGAATCCATTCTCACTTCTCGATTATTTTACCTCACTATTTT**A**TTTCT

TCATGAACAGAA**T**AAATTAGAACATGAATCTGTCCATCCATCTTATGACAAGTTGAGTTG

ATCAGT

LF; Aa_trnLF-for, Aa_trnLF-rev; A+G (haplotype 9); 186 bases

GGTTCAAGTCCCTCTATCCCCACCTAGGTTCGTTCCCGAACGACTGATCTATTTTCTCCA

ATTCCATTAGTTCGAATCCATTCTCACTTCTCGATTATTTTACCTCACTATTTT**A**TTTCT

TCATGAACAGAA**G**AAATTAGAACATGAATCTGTCCATCCATCTTATGACAAGTTGAGTTG

ATCAGT

K2i; trnK2i-f1, trnK2i-r1; C (haplotype 1-3, 5-9, and cone, S9, S10); 204 bases

GCCCTCGTTCATGAGAATAACCTCTTAAATTCTGAGATAATACAATACATGGTGCGATCT

AGTCGGGACAAGATAGGAAAAAGATAGATGATATAAAGATTCTATCTTCTATT**C**AGCAGA

TTTACTACCCAAGGATCTCGTTCGTCATGAGGAAGAACGAAAATCTTTTATCCTGGCAAC

CAATCGCTCTCCTGACTCATGACG

K2i; trnK2i-f1, trnK2i-r1; T (haplotype 4); 204 bases

GCCCTCGTTCATGAGAATAACCTCTTAAATTCTGAGATAATACAATACATGGTGCGATCT

AGTCGGGACAAGATAGGAAAAAGATAGATGATATAAAGATTCTATCTTCTATT**T**AGCAGA

TTTACTACCCAAGGATCTCGTTCGTCATGAGGAAGAACGAAAATCTTTTATCCTGGCAAC

CAATCGCTCTCCTGACTC
