# Supplementary material for: Population dynamics and genetic changes of Picea abies in the South Carpathians revealed by pollen and ancient DNA analyses
Source: BMC Evol Biol. 2011 Mar 10;11:66. doi: 10.1186/1471-2148-11-66 (PMC3068097; doi:10.1186/1471-2148-11-66)
Supplement: Additional file 2 — Radiocarbon dates and age-depth modeling of sediment core TDB-1, Taŭl dintre Brazi (1740 m a.s.l), Retezat Mountains, South Carpathians, Romania. [file 1471-2148-11-66-S2.DOC]

**Additional file 2 - Radiocarbon dates and age-depth modeling of sediment core TDB-1, Taŭl dintre Brazi (1740 m a.s.l), Retezat Mountains, South Carpathians, Romania.**

Radiocarbon dates from Taŭl dintre Brazi (TDB-1). AMS ^14^C dates were obtained from the Poznań Radiocarbon Laboratory, Poland.

| Core | Laboratory  code | Dated material | Depth  (cm) | ^14^C age  years BP | Calibrated range  years BP (2σ) |
| --- | --- | --- | --- | --- | --- |
| TDB-1 | Poz-26103 | *Picea abies* needles | 119 | 725 ± 30 | 652-723 |
| TDB-1 | Poz-26104 | *Pinus mugo* cone scale | 160 | 1735 ± 30 | 1562-1712 |
| TDB-1 | Poz-26106 | *Pinus mugo* cone | 238 | 3045 ± 30 | 3205-3356 |
| TDB-1 | Poz-26107 | *Pinus* twig | 315 | 5040 ± 40 | 5708-5902 |
| TDB-1 | Poz-26108 | *Picea abies* needles | 355 | 6320 ± 40 | 7163-7324 |
| TDB-1 | Poz-26110 | *Picea abies* seed *&* needles | 450 | 8240 ± 50 | 9072-9326 |
| TDB-1 | Poz-26111 | *Picea abies* needles | 505 | 8810 ± 50 | 9670-9966 |
| TDB-1 | Poz-31714 | *Pinus mugo* needles | 521 | 9150 ± 50 | 10223-10432 |
| TDB-1 | Poz-26112 | *Picea abies* cone | 545 | 9610 ± 50 | 10764-11165 |
| TDB-1 | Poz-31715 | *Pinus mugo* needles | 557 | 9980 ± 100 | 11216-11618 |
| TDB-1 | Poz-31716 | charcoal | 569 | 10870 ± 70 | 12598-12925 |
| TDB-1 | Poz-27305 | *Pinus sp*. needles (2) | 578 | 11590 ± 60 | 13287-13620 |

Non-linear weighted polynomial regression for modeling age-depth relationship in the lateglacial and early Holocene part of TDB-1. The 3D image displays the regression line and the posterior probability distributions of the ^14^C dates**.**

Linear age-depth model for TDB-1 based on 12 ^14^C dates, calibration using BCal and age-model production using linear interpolation in psimpoll. Thick gray horizontal lines indicate the posterior probability distribution of the radiocarbon dates and the middle vertical lines indicate the mode of posterior probability distribution; solid line indicates the psimpoll age model. Note that the final age-depth model used on Figures 5 and 6 combines the linear and non-linear weighted polynomial regression models. Below 10,000 cal yr BP the polynomial model is used, while above 10,000 cal yr BP the linear model is used. For further details see Magyari et al. [43]
