# Supplementary material for: Population dynamics and genetic changes of Picea abies in the South Carpathians revealed by pollen and ancient DNA analyses
Source: BMC Evol Biol. 2011 Mar 10;11:66. doi: 10.1186/1471-2148-11-66 (PMC3068097; doi:10.1186/1471-2148-11-66)
Supplement: Additional file 3 — Location and geographical coordinates of the extant Norway spruce (Picea abies) samples and herbarium specimens used for cpDNA analysis. [file 1471-2148-11-66-S3.DOC]

**Additional file 3 - Location and geographical coordinates of the extant Norway spruce (*Picea* *abies*) samples and herbarium specimens used for cpDNA analysis**

| Sample  name | Location | Haplo-type | Collection  date | Latitude N | Longitude E | Altitude  (m) |
| --- | --- | --- | --- | --- | --- | --- |
| CP2 | Retezat Mts, Romania | Ht 3 | 2008 | 45.431833 | 22.894850 | 1065 |
| CP3 | Retezat Mts, Romania | Ht 2 | 2008 | 45.430700 | 22.894533 | 1068 |
| CP6 | Retezat Mts, Romania | Ht 2 | 2008 | 45.422150 | 22.891783 | 1162 |
| CP8 | Retezat Mts, Romania | Ht 8 | 2008 | 45.412050 | 22.886950 | 1292 |
| CP10 | Retezat Mts, Romania | Ht 8 | 2008 | 45.407733 | 22.883617 | 1427 |
| St2 | Retezat Mts, Romania | Ht 5 | 2008 | 45.402117 | 22.872283 | 1584 |
| St6 | Retezat Mts, Romania | Ht 1 | 2008 | 45.397283 | 22.864783 | 1721 |
| St10 | Retezat Mts, Romania | Ht 3 | 2008 | 45.394667 | 22.863917 | 1762 |
| St15 | Retezat Mts, Romania | Ht 7 | 2008 | 45.395667 | 22.864017 | 1751 |
| St19 | Retezat Mts, Romania | Ht 3 | 2008 | 45.398967 | 22.866483 | 1705 |
| P5 | Retezat Mts, Romania | Ht 1 | 2008 | 45.398333 | 22.881111 | 1565 |
| P9 | Retezat Mts, Romania | Ht 6 | 2008 | 45.390833 | 22.880278 | 1702 |
| P14 | Retezat Mts, Romania | Ht 8 | 2008 | 45.388889 | 22.881111 | 1723 |
| P16 | Retezat Mts, Romania | Ht 2 | 2008 | 45.386944 | 22.880278 | 1774 |
| P18 | Retezat Mts, Romania | Ht 8 | 2008 | 45.386389 | 22.880556 | 1781 |
| R3 | Retezat Mts, Romania | Ht 1 | 2008 | 45.399617 | 22.883850 | 1535 |
| R6 | Retezat Mts, Romania | Ht 2 | 2008 | 45.397500 | 22.889250 | 1700 |
| R9 | Retezat Mts, Romania | Ht 3 | 2008 | 45.394867 | 22.889150 | 1811 |
| R11 | Retezat Mts, Romania | Ht 8 | 2008 | 45.394283 | 22.888250 | 1840 |
| R12 | Retezat Mts, Romania | Ht 6 | 2008 | 45.393917 | 22.887850 | 1851 |
| Gtk4 | Retezat Mts, Romania | Ht 8 | 2008 | 45.406440 | 22.890360 | 1740 |
| Gtk8 | Retezat Mts, Romania | Ht 8 | 2008 | 45.401770 | 22.900040 | 1740 |
| Gtk9 | Retezat Mts, Romania | Ht 2 | 2008 | 45.400590 | 22.901750 | 1740 |
| Gt13 | Retezat Mts, Romania | Ht 1 | 2008 | 45.397170 | 22.902590 | 1740 |
| Gt14 | Retezat Mts, Romania | Ht 8 | 2008 | 45.396660 | 22.901890 | 1740 |
| Gt15 | Retezat Mts, Romania | Ht 2 | 2008 | 45.396510 | 22.901820 | 1740 |
| Gt17 | Retezat Mts, Romania | Ht 2 | 2008 | 45.396410 | 22.901330 | 1740 |
| Gt18 | Retezat Mts, Romania | Ht 1 | 2008 | 45.396710 | 22.901150 | 1740 |
| Gt20 | Retezat Mts, Romania | Ht 2 | 2008 | 45.396900 | 22.901500 | 1740 |
| Gtk22 | Retezat Mts, Romania | Ht 1 | 2008 | 45.396906 | 22.905086 | 1740 |
| Gtk24 | Retezat Mts, Romania | Ht 4 | 2008 | 45.396470 | 22.905310 | 1740 |
| B3 | Retezat Mts, Romania | Ht 8 | 2008 | 45.306944 | 22.981111 | 1085 |
| B11 | Retezat Mts, Romania | Ht 1 | 2008 | 45.307778 | 22.948056 | 1163 |
| B15 | Retezat Mts, Romania | Ht 8 | 2008 | 45.311944 | 22.923889 | 1208 |
| B21 | Retezat Mts, Romania | Ht 8 | 2008 | 45.318333 | 22.914722 | 1342 |
| B25 | Retezat Mts, Romania | Ht 8 | 2008 | 45.323056 | 22.913611 | 1429 |
| M2 | Retezat Mts, Romania | Ht 8 | 2008 | 45.304200 | 22.978817 | 1132 |
| M5 | Retezat Mts, Romania | Ht 8 | 2008 | 45.317933 | 22.972367 | 1117 |
| M15 | Retezat Mts, Romania | Ht 8 | 2008 | 45.328533 | 22.953467 | 1443 |
| M20 | Retezat Mts, Romania | Ht 3 | 2008 | 45.333133 | 22.949683 | 1622 |
| M25 | Retezat Mts, Romania | Ht 8 | 2008 | 45.337767 | 22.948950 | 1792 |
| Z1 | Retezat Mts, Romania | Ht 2 | 2008 | 45.310000 | 22.983233 | 928 |
| Z7 | Retezat Mts, Romania | Ht 8 | 2008 | 45.317633 | 22.990067 | 1147 |
| Z14 | Retezat Mts, Romania | Ht 8 | 2008 | 45.326100 | 22.989933 | 1432 |
| Z22 | Retezat Mts, Romania | Ht 8 | 2008 | 45.341017 | 22.991600 | 1718 |
| Z26 | Retezat Mts, Romania | Ht 2 | 2008 | 45.348033 | 22.991267 | 1912 |
| Buk9 | Retezat Mts, Romania | Ht 2 | 2008 | 45.341731 | 22.889435 | 2041 |
| Buk12-3 | Retezat Mts, Romania | Ht 3 | 2008 | 45.347390 | 22.883125 | 2041 |
| Buk14-1 | Retezat Mts, Romania | Ht 8 | 2008 | 45.351734 | 22.879741 | 2041 |
| KR6-1 | Retezat Mts, Romania | Ht 8 | 2008 | 45.333150 | 22.897900 | 1792 |
| KR8-2 | Retezat Mts, Romania | Ht 5 | 2008 | 45.284117 | 22.846967 | 1921 |
| H5 | Jaszinya,Ukraine | Ht 2 | 1940 | 48.258889 | 24.355000 |  |
| H6 | Bihor Mts, Romania | Ht 2 | 1882 | 46.657242 | 22.677084 |  |
| H9 | Chotča, Slovakia | Ht 8 | 1981 | 49.241667 | 21.678611 |  |
| H10 | Studená dolina, Vysoké Tatry, Slovakia | Ht 2 | 1890 | 49.138889 | 20.219444 |  |
| H11 | Králova holá, Nízke Tatry, Slovakia | Ht 2 | 1906 | 49.106111 | 21.731944 |  |
| H12 | Rožňava-Jasov, Slovakia | Ht 9 | 1976 | 48.604793 | 20.660660 |  |
| AF | Alps, Austria | Ht 3 | 2009 | 46.970343 | 12.960239 |  |
